# Combining the Fibrinogen/Albumin Ratio and Systemic Inflammation Response Index Predicts Survival in Resectable Gastric Cancer

**DOI:** 10.1155/2020/3207345

**Published:** 2020-02-25

**Authors:** Junbin Zhang, Yongfeng Ding, Weibin Wang, Yimin Lu, Haiyong Wang, Haohao Wang, Lisong Teng

**Affiliations:** ^1^Department of Surgical Oncology, The First Affiliated Hospital, College of Medicine, Zhejiang University, Qingchun Road 79, Hangzhou, China; ^2^Cancer Center, The First Affiliated Hospital, School of Medicine, Zhejiang University, Qingchun Road 79, Hangzhou, China

## Abstract

**Aims:**

Predicting the prognosis of gastric cancer using tumour-node-metastasis (TNM) staging is difficult as patients with the same TNM stage exhibit different prognoses.

**Methods:**

This study investigated the prognostic value of the preoperative fibrinogen/albumin ratio (FAR)-systemic inflammation response index (SIRI) score in resectable gastric cancer (rGC).

**Results:**

Clinicopathological features of 231 rGC patients were analysed retrospectively. Patients were divided into three groups: FAR-SIRI score 2 (FAR ≥ 0.071 and SIRI ≥ 0.84), 1 (FAR < 0.071 and SIRI ≥ 0.84), and 0 (SIRI < 0.84). Higher FAR-SIRI scores were associated with larger tumours, poorer differentiation, and advanced TNM stage (*P* < 0.05). Compared to those with FAR-SIRI scores of 0, patients with scores of 2 had poorer overall survival (OS). The FAR-SIRI score was an independent prognostic factor for OS in rGC.

**Conclusion:**

The present data demonstrated that FAR-SIRI scores predicted radical gastric cancer surgical outcomes and may serve as a blood marker for identifying high-risk patients.

## 1. Introduction

Gastric cancer is the second most prevalent malignant cancer, with the third highest mortality rate, and it is characterized by occult development, a low early diagnosis rate, and poor prognoses [1]. At present, the treatment strategies and prognosis of gastric cancer are mainly based on tumour-node-metastasis (TNM) staging. However, accurate TNM staging can only be performed after surgery, and gastric cancer patients with the same TNM stage may show different prognoses. Therefore, there is an urgent need to identify a simple and feasible prognostic index for gastric cancer that can assist in prognostic estimation at the time of diagnosis, thus facilitating the overall comprehensive clinical management of patients.

Inflammation is closely related to tumour progression and prognosis. Over the past few decades, immune cell-related indices such as the neutrophil-to-lymphocyte ratio (NLR), platelet-to-lymphocyte ratio (PLR), and lymphocyte-to-monocyte ratio (LMR) have been used to predict prognosis and recurrence in various malignancies [2]. In recent years, a novel inflammatory marker, the systemic inflammation response index (SIRI), which is calculated using the levels of neutrophils, monocytes, and lymphocytes, has shown an ability to predict prognosis in pancreatic cancer [3], gastric cancer [4], and nasopharyngeal cancer [5].

In addition to inflammation, abnormal coagulation and nutritional status are also related to malignant tumour occurrence and development. Many studies have shown that increased plasma fibrinogen levels are associated with poor prognosis in gastric cancer, non-small-cell lung cancer, and renal cancer [6–8]. Albumin is an important indicator of nutritional status and commonly used for nutritional status assessment. Most patients with advanced tumours, including those with gastric cancer, show decreased albumin levels. Therefore, as a newly discovered marker, the fibrinogen/albumin ratio (FAR) in combination with a patient's coagulation and nutritional status can predict prognosis in soft tissue tumours [9], liver cancer [10], and oesophageal cancer [11].

Here, we proposed the use of a novel marker, the FAR-SIRI score, in combination with inflammation, coagulation function, and nutritional status and investigated its prognostic impact in patients with resectable gastric cancer.

## 2. Materials and Methods

### 2.1. Patients and Follow-Up

We performed a retrospective study of patients with gastric cancer confirmed by histopathology at the First Affiliated Hospital, College of Medicine, Zhejiang University (Hangzhou, China) from April 2012 to January 2016. The ethics board approved the research protocols of the present study, which followed the tenets of the Declaration of Helsinki. Written informed consent was obtained from the individual patients. Data on the following clinicopathological characteristics were collected from medical records: sex, age, primary tumour location, differentiation, tumour size, and TNM stage. Similar to other studies [12–14], blood samples were obtained within 1 week before surgery for the measurement of neutrophil, lymphocyte, monocyte, platelet, albumin (Alb), and fibrinogen (Fib) levels. Patients meeting any of the following criteria were excluded: (1) histologically confirmed stage IV disease, (2) severe complications or death within 15 days after diagnosis, (3) incomplete pretreatment laboratory parameters, (4) malignancies other than gastric cancer, (5) haematological disorders which could potentially affect the white blood cells, and (6) evidence of any autoimmune or infectious diseases or liver dysfunction indicated by abnormal alanine aminotransferase. This study was approved by the Institutional Review Board of the First Affiliated Hospital, College of Medicine, Zhejiang University. TNM stage was assessed based on guidelines in the eighth edition of the American Joint Committee on Cancer staging. Patients were followed up carefully after surgery at 3- to 6-month intervals. Overall survival (OS) was calculated from the date of surgery to the date of death or last follow-up. In our study, the final follow-up was conducted in March 2019.

### 2.2. Definition of Inflammation-Based Indicators and Optimal Cut-off Calculation

The NLR and PLR were defined as the absolute neutrophil count and platelet count divided by the absolute lymphocyte count, respectively. The SIRI was calculated as follows: SIRI = N × M/L, where N, M, and L signify the preoperative neutrophil, monocyte, and lymphocyte counts, respectively. The FAR was defined as follows: FAR = F/A, where F and A represent the preoperative Fib and Alb levels, respectively. The optimal cut-off neutrophil, lymphocyte, monocyte, and platelet counts and Alb, Fib, NLR, PLR, FAR, and SIRI values were computed using receiver operating characteristic (ROC) curves. In the current study, the FAR-SIRI score was determined by a combination of the FAR and SIRI scores.

### 2.3. Statistical Analysis

Categorical variables were analysed using the chi-square test or Fisher's exact test. The optimal cut-off for each indicator was calculated by ROC curve analysis. The estimated area under the curve (AUC) was calculated as the prognostic ability of each variable. Kaplan-Meier curves were compared using the log-rank test. The univariate and multivariate logistic regression tests were used to determine the independent effect of clinicopathological features. Statistical analyses were conducted using SAS 9.4 (SAS Institute, Inc., Cary, NC, USA) and the survival ROC and time ROC [15] packages in R version 3.3.0. In this study, all *P* values were two-sided, and *P* values < 0.05 were considered statistically significant.

## 3. Results and Discussion

### 3.1. Clinicopathological Characteristics

Overall, 231 patients were enrolled in our study; their clinicopathological and laboratory characteristics are shown in Table 1. The enrolled cohort comprised 156 (67.5%) men and 75 (32.5%) women with a median age of 62 years (range, 26–85 years). Based on the eighth American Joint Committee on Cancer TNM staging system, 59 (25.5%), 65 (28.1%), and 107 (46.3%) patients were diagnosed as having stages I, II, and III, respectively. The median follow-up period was 43 months (range, 3–73 months). The optimal cut-off values for all the inflammation-based indicators were calculated using ROC curves, as shown in Table 1.

### 3.2. Evaluation of Prognostic Abilities for Inflammation-Based Indicators

**Figure 1:**
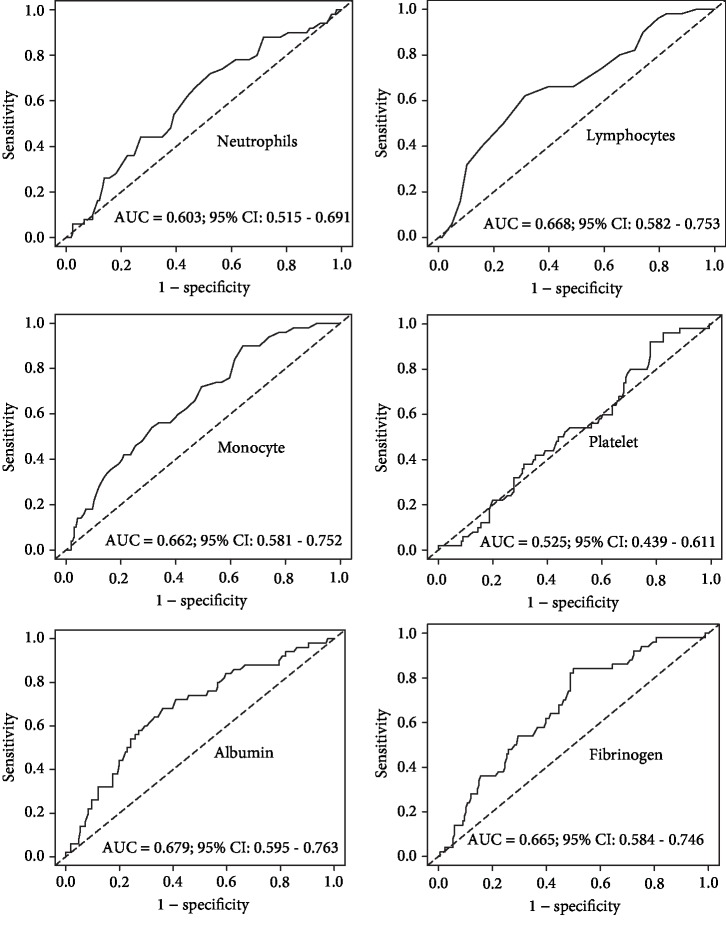
The predictive ability of the neutrophil, lymphocyte, monocyte, platelet, albumin, and fibrinogen levels was calculated by ROC curves. AUC: area under the curve; CI: confidence interval.

**Figure 2:**
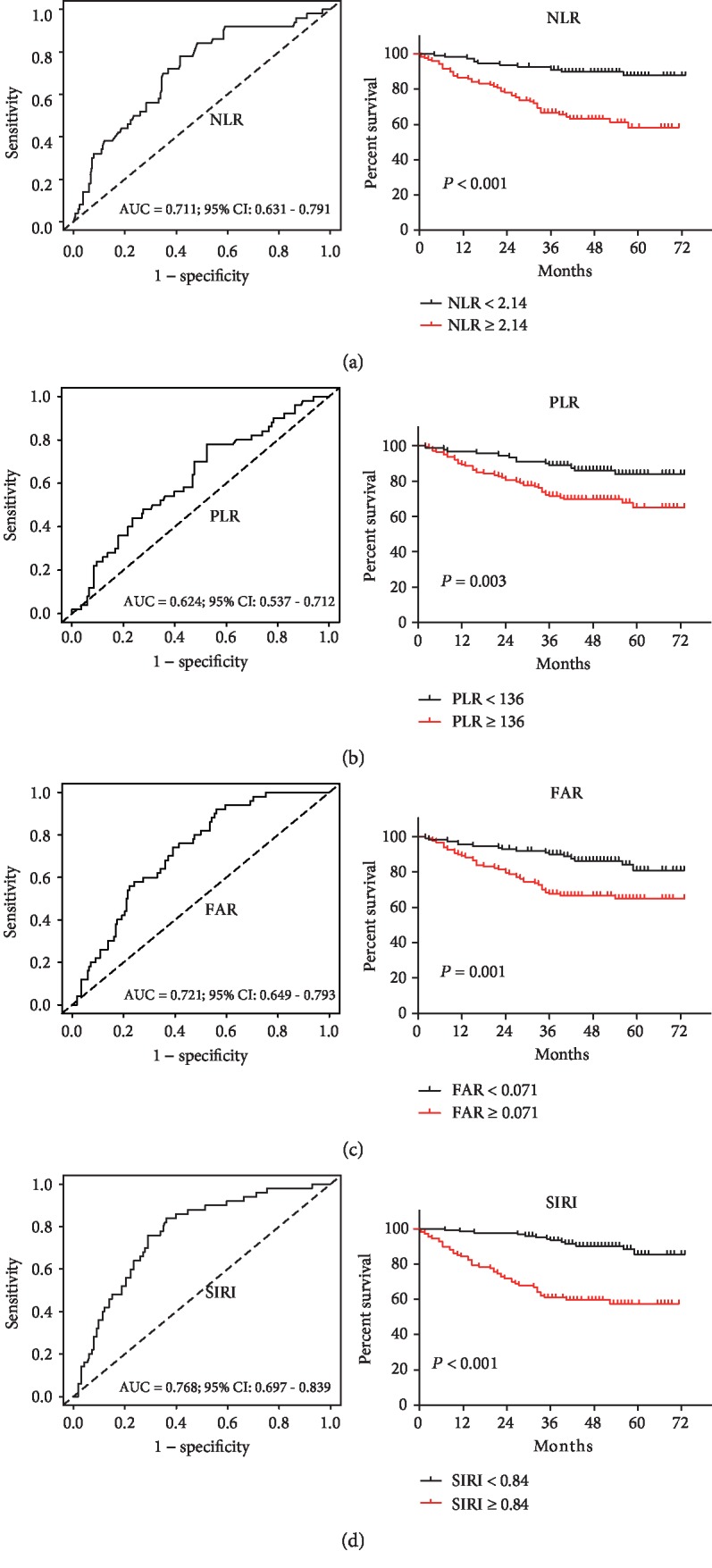
Receiver operating characteristic curve (ROC) analyses of optimal cutoffs and survival analysis for (a) NLR, (b) PLR, (c) FAR, and (d) SIRI in patients with GC. NLR: neutrophil-lymphocyte ratio; PLR: platelet-lymphocyte ratio; FAR: fibrinogen-albumin ratio; SIRI = N × M/L, where N, M, and L represent the preoperative counts of neutrophils, monocytes, and lymphocytes, respectively. Kaplan-Meier survival analysis with the log-rank test was used to calculate *P* values.

The prognostic abilities of the inflammation-based indicators were calculated by ROC curve generation and AUC estimation. The AUC values for the neutrophil, lymphocyte, monocyte, platelet, Alb, and Fib levels are shown in Figure 1. We computed the AUC values for the NLR (AUC = 0.711; 95% confidence interval [CI] = 0.631‐0.791), PLR (AUC = 0.624; 95%CI = 0.537‐0.712), FAR (AUC = 0.721; 95%CI = 0.649‐0.793), and SIRI (AUC = 0.768; 95%CI = 0.697‐0.839) (left panel Figure 2). In addition, based on the calculated optimal cut-offs (2.14 for the NLR, 136 for the PLR, 0.071 for the FAR, and 0.84 for the SIRI), we performed corresponding survival analyses (right panel Figure 2, all *P* < 0.01).

### 3.3. Establishment of the FAR-SIRI Score

**Figure 3:**
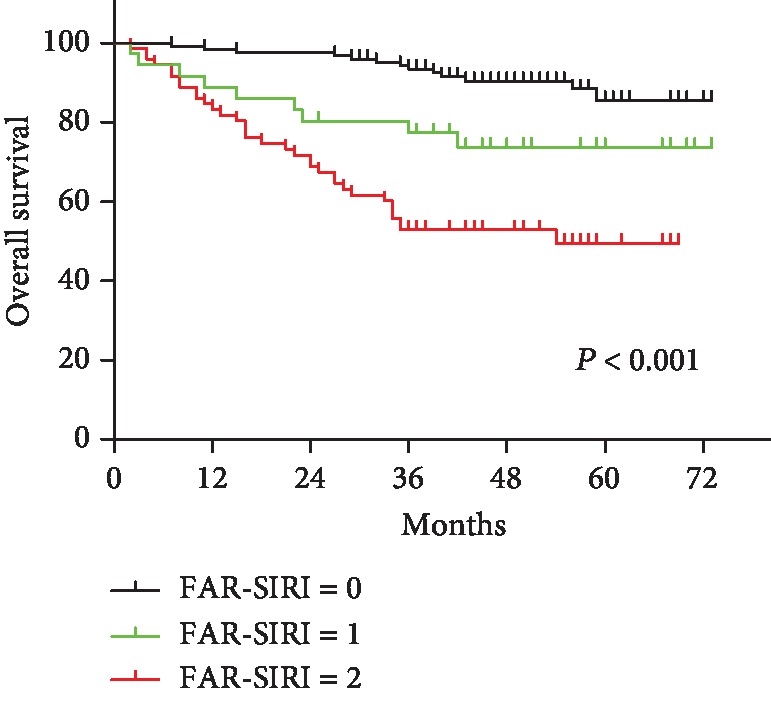
Effects of recombination of FAR and SIRI (FAR-SIRI score) on the survival of GC patients. FAR-SIRI score 0 represents patients with SIRI < 0.84; FAR-SIRI score 1 represents patients with FAR score < 0.071 and SIRI score ≥ 0.84. Patients with FAR score ≥ 0.071 and SIRI score ≥ 0.84 were assigned a FAR-SIRI score of 2. *P* value was calculated by the log-rank test.

Based on the aforementioned results, patients with a FAR score ≥ 0.071 and a SIRI score ≥ 0.84 were assigned a score FAR-SIRI of 2, patients with a FAR score < 0.071 and a SIRI score ≥ 0.84 were assigned a FAR-SIRI score of 1, and patients with a SIRI < 0.84 were assigned a FAR-SIRI score of 0, regardless of the FAR score. Based on the FAR-SIRI system, 123 (53.2%), 36 (15.6%), and 72 (31.2%) of the patients had scores of 0, 1, and 2, respectively. As shown in Figure 3, gastric cancer patients with higher FAR-SIRI values had worse prognoses.

The relationship between FAR-SIRI scores and clinicopathological factors is presented in Table 2. Elevated FAR-SIRI scores were more likely to be observed in men (*P* = 0.030) and were associated with tumour location (upper third) (*P* = 0.010), larger tumour size (*P* < 0.001), poor differentiation (*P* = 0.006), and higher T stage, N stage, and TNM stage (*P* < 0.001 for all).

### 3.4. The FAR-SIRI Score Independently Predicts OS

As shown in Table 3, age (hazard ratio [HR] = 2.979, 95%CI = 1.461‐6.073, *P* = 0.003), tumour location (HR = 0.530, 95%CI = 0.281‐0.998, *P* = 0.049 for lower third vs. upper third), tumour size (HR = 3.615, 95%CI = 1.773‐7.370, *P* < 0.001), TNM stage (HR = 17.261, 95%CI = 4.190‐71.105, *P* < 0.001 for TNM stage III vs. I), and FAR-SIRI score (HR = 2.548, 95%CI = 1.103‐5.890, *P* = 0.029 for 1 vs. 0; HR = 5.760, 95%CI = 3.082‐10.763, *P* < 0.001 for 2 vs. 0) were significantly associated with OS in the univariate analysis. Multivariate analysis revealed that age (HR = 2.313; 95%CI = 1.074‐4.981; *P* = 0.032), tumour differentiation (HR = 2.209; 95%CI = 1.009‐4.835; *P* = 0.048), TNM stage (HR = 9.893, 95%CI = 2.029‐48.236, *P* = 0.005 for stage III vs. I), and FAR-SIRI score (HR = 2.718, 95%CI = 1.372‐5.386, *P* = 0.004 for 2 vs. 0) were independent prognostic indicators in gastric cancer patients.

## 4. Discussion

At present, the TNM staging system is still the gold standard for the prognostic assessment and treatment of various malignancies. The results of this study show that the FAR-SIRI is an independent prognosticator in gastric cancer. The FAR-SIRI is a simple, convenient, inexpensive indicator that can supplement TNM staging in identification of high-risk patients. Since TNM staging can only be obtained after surgery, predicting prognosis before operation can make more individualized treatment plan for some patients.

Inflammation is among the hallmarks of malignancy. Tumour inflammatory microenvironments are complex and dynamic, involving crosstalk between various immune cells and tumour cells. Inflammatory prognostic scores such as the NLR, PLR, and LMR calculated using immune cell-related values have shown promise in various tumour types [16–19]. In addition, the efficacy of the SIRI has been reported in limited types of cancer, such as liver [20], pancreatic [3], gastric [4], and nasopharyngeal [5] cancers. Our study also confirmed that it can be used as a prognostic indicator in gastric cancer patients undergoing radical gastrectomy, i.e., a high SIRI indicates poor prognosis.

The SIRI reflects the complex interaction and synergistic promotion among the principal immune cells (neutrophils, lymphocytes, and monocytes) in the cancer microenvironment. Neutrophils are the most prevalent immune cells in human peripheral blood. They can promote tumour growth and metastasis through the release of a variety of inflammatory factors or mechanisms, such as neutrophil extracellular trapping [21, 22]. In addition, neutrophils can also inhibit the anti-tumour immunity of cytotoxic T cells and NK cells [23]. Activated neutrophils can produce vascular endothelial growth factor and promote tumour angiogenesis [24]. Lymphocytes play an essential role in tumour-related immunity, especially antitumour immunity. Elevated lymphocyte levels are positively correlated with prognosis in various cancers [25, 26]. Lymphocytes secrete various cytokines, such as interferon-*γ* and tumour necrosis factor-*α*, thus controlling tumour growth and improving prognosis [27]. Decreases in the lymphocyte function, degree, or number can weaken a tumour's immune surveillance and defence ability [28]. The presence of mononucleosis indicates relatively poor prognosis in various cancers. Monocytes can infiltrate tumours and differentiate into tumour-related macrophages, which can promote the growth, invasion, and migration of tumours, as well as induce apoptosis in activated CD8 T cells, which have anticancer activity [29].

In recent years, several studies have found that the hypercoagulable state is related to malignant tumour progression [30]. Fibrinogen is not only an important component in the coagulation cascade but also an inflammation-related acute reactive protein. Fibrinogen plays a major role in tumour-related biological behaviours such as cell proliferation, epithelial-mesenchymal transformation, and angiogenesis [31, 32]. It can also provide a stable framework for the tumour extracellular matrix, thus promoting cancer cell adhesion, migration, and invasion [33].

Albumin is the most abundant protein in plasma, accounting for about 50% of the total protein content. Decreases in albumin are reflective of malnutrition, suggesting that the immune ability is weakened, leading to an increased risk of infection and tumour progression, which is related to poor tumour-related prognosis [10]. Preoperative albumin levels have prognostic significance in renal cancer [34], head and neck cancer [35], and ovarian cancer [36].

Although it has been reported that the FAR predicts prognosis in malignancy, to the best of our knowledge, our study is the first to report on its prognostic role in resectable gastric cancer.

Various indicators such as the NLR, PLR, and LMR can predict the prognosis in gastric cancer patients undergoing radical gastrectomy [37, 38]. Some studies have also found that combining two indicators, such as fibrinogen and the NLR, is better able to predict prognosis in various tumours, including gastric cancer [39]. The FAR-SIRI score, as proposed in this study, combines systemic inflammation, coagulation function, and nutritional status to analyse the pathophysiological conditions of malignant tumours. As shown in Figure 3, we introduced the FAR score into the SIRI score to create a new prognostic indicator and found that the FAR-SIRI score can be used to effectively divide patients into three different risk groups. Moreover, high FAR-SIRI scores were associated with larger tumour size, deeper infiltration, and lymph node metastasis, supporting the hypothesis that this indicator is associated with tumour invasion and metastasis. Clinicians should pay more attention and provide appropriate intervention to patients with a FAR-SIRI score of 2. The evaluation should be emphasized during the postoperative chemotherapy and we tend to choose more aggressive chemotherapy scheme for the patients with high FAR-SIRI score.

Our study has some limitations. First, it used a single-centre retrospective design, which is associated with certain bias; therefore, our findings need to be confirmed in a multicentre study. Second, differences in the patients' postoperative treatments/chemotherapy types may lead to confusions with regard to the results. Although OS is the standard detection index used for the judgment of prognosis in cancer, we lacked data on disease-free survival. Similarly, several studies evaluated the prognostic value of hematological pretreatment parameters on gastric cancer by only using OS [39–41]; therefore, our conclusion needs to be confirmed by other survival measures, such as disease-free survival.

## 5. Conclusions

In conclusion, the FAR-SIRI score is a convenient, inexpensive, reliable marker that can be used as a screening and prognostic indicator for high-risk patients with gastric cancer, providing a reference for long-term management and treatment after surgery.

## Figures and Tables

**Table 1:** Clinical and laboratory characteristics of 231 GC patients.

	No. of patients (%)
Sex	
Male	156 (67.5)
Female	75 (32.5)
Age (years)	
<60	76 (32.9)
≥60	155 (67.1)
Neutrophils (×10^9^/L)	
<3	90 (39.0)
≥3	141 (61.0)
Lymphocytes (×10^9^/L)	
<1.4	89 (38.5)
≥1.4	142 (61.5)
Monocyte (×10^9^/L)	
<0.29	49 (21.2)
≥0.29	182 (78.8)
Platelet (×10^9^/L)	
<237	135 (58.4)
≥237	96 (41.6)
Alb (g/L)	
<40.8	92 (39.8)
≥40.8	139 (60.2)
FIB (g/L)	
<2.79	91 (39.4)
≥2.79	140 (60.6)
NLR	
<2.14	111 (48.1)
≥2.14	120 (51.9)
PLR	
<136	91 (39.4)
≥136	140 (60.6)
FAR	
<0.071	112 (48.5)
≥0.071	119 (51.5)
SIRI	
<0.84	124 (53.7)
≥0.84	107 (46.3)
Tumour location	
Upper third	54 (23.4)
Middle third	53 (22.9)
Lower third	119 (51.5)
Mixed	5 (2.2)
Tumour size (cm)	
<3	86 (37.2)
≥3	145 (62.8)
Differentiation	
Poor	194 (84.0)
Moderate and well	37 (16)
TNM stage	
I	59 (25.5)
II	65 (28.1)
III	107 (46.3)

**Table 2:** The clinicopathological characteristics stratified by the FAR-SIRI score.

Characteristics	FAR-SIRI 0	FAR-SIRI 1	FAR-SIRI 2	*P* value
	(*n* = 123)	(*n* = 36)	(*n* = 72)	
Sex				
Male	74 (47.4)	29 (18.6)	53 (34.0)	0.030
Female	49 (65.3)	7 (9.3)	19 (25.3)	
Age (years)				
<60	48 (63.2)	10 (13.2)	18 (23.7)	0.103
≥60	75 (48.4)	26 (16.8)	54 (34.8)	
Tumour location				
Upper third	22 (40.7)	14 (25.9)	18 (33.3)	0.010^∗^
Middle third	35 (66.0)	6 (11.3)	12 (22.6)	
Lower third	66 (55.5)	15 (12.6)	38 (31.9)	
Mixed	0 (0)	1 (20)	4 (80)	
Tumour size (cm)				
<3	64 (74.4)	11 (12.8)	11 (12.8)	<0.001
≥3	59 (40.7)	25 (17.2)	61 (42.1)	
T stage				
1	45 (95.7)	1 (2.1)	1 (2.1)	<0.001
2	20 (64.5)	6 (19.4)	5 (16.1)	
3	31 (42.5)	13 (17.8)	29 (39.7)	
4	27 (33.8)	16 (20.0)	37 (46.3)	
N stage				
1	64 (78.1)	8 (9.8)	10 (12.2)	<0.001
2	23 (46.9)	13 (26.5)	13 (26.5)	
3	20 (30.8)	10 (15.4)	35 (53.9)	
Differentiation				
Poor	95 (49.0)	35 (18.0)	64 (33.0)	0.006
Moderate and well	28 (75.7)	1 (2.7)	8 (21.6)	
TNM stage				
I	54 (91.5)	4 (6.8)	1 (1.7)	<0.001
II	35 (53.9)	11 (16.9)	19 (29.2)	
III	34 (31.8)	21 (19.6)	52 (48.6)	

∗Fisher's exact test; others, chi-square test.

**Table 3:** Univariate and multivariate analysis of OS clinicopathologic variables in relation to OS in resectable gastric cancer patients.

	Univariate analysis		Multivariate analysis	
	HR (95% CI)	*P* value	HR (95% CI)	*P* value
Sex				
Male	Ref	0.085	Ref	0.939
Female	0.581 (0.313, 1.078)		0.974 (0.494, 1.918)	
Age (years)				
<60	Ref	0.003	Ref	0.032
≥60	2.979 (1.461, 6.073)		2.313 (1.074, 4.981)	
Tumour location				
Upper third	Ref		Ref	
Middle third	0.697 (0.338, 1.435)	0.327	1.227 (0.584, 2.574)	0.589
Lower third	0.53 (0.281, 0.998)	0.049	0.664 (0.341, 1.294)	0.229
Tumour size (cm)				
<3	Ref		Ref	
≥3	3.615 (1.773, 7.370)	<0.001	1.295 (0.598, 2.805)	0.512
Differentiation				
Poor	Ref		Ref	
Moderate and well	0.938 (0.460, 1.913)	0.861	2.209 (1.009, 4.835)	0.048
TNM stage				
I	Ref		Ref	
II	3.911 (0.831, 18.412)	0.085	2.516 (0.502, 12.602)	0.262
III	17.261 (4.190, 71.105)	<0.001	9.893 (2.029, 48.236)	0.005
FAR-SIRI				
0	Ref		Ref	
1	2.548 (1.103, 5.890)	0.029	1.229 (0.493, 3.064)	0.658
2	5.760 (3.082, 10.763)	<0.001	2.718 (1.372, 5.386)	0.004

## Data Availability

The datasets used and/or analyzed during the present study are available from the corresponding author on reasonable request.
